# Virtual Reality for Enhancing the Cognitive Behavioral Treatment of Obesity With Binge Eating Disorder: Randomized Controlled Study With One-Year Follow-up

**DOI:** 10.2196/jmir.2441

**Published:** 2013-06-12

**Authors:** Gian Luca Cesa, Gian Mauro Manzoni, Monica Bacchetta, Gianluca Castelnuovo, Sara Conti, Andrea Gaggioli, Fabrizia Mantovani, Enrico Molinari, Georgina Cárdenas-López, Giuseppe Riva

**Affiliations:** ^1^Applied Technology for Neuro Psychology LabIstituto Auxologico ItalianoVerbaniaItaly; ^2^Centro Obesità e Nutrizione Clinica (CONC)Ospedale Privato Accreditato Villa IgeaForlì (FC)Italy; ^3^Psychology Research LaboratoryOspedale San GiuseppeIstituto Auxologico ItalianoVerbaniaItaly; ^4^Department of PsychologyCatholic University of Sacred HeartMilanItaly; ^5^CESCOM-Centre for Research in Communication SciencesUniversity of Milan-BicoccaMilanItaly; ^6^Laboratorio de Enseñanza Virtual y CiberpsicologíaFacultad de PsicologíaUniversidad Nacional Autónoma de MéxicoMéxicoMexico

**Keywords:** virtual reality, obesity, binge eating disorders, allocentric lock hypothesis

## Abstract

**Background:**

Recent research identifies unhealthful weight-control behaviors (fasting, vomiting, or laxative abuse) induced by a negative experience of the body, as the common antecedents of both obesity and eating disorders. In particular, according to the allocentric lock hypothesis, individuals with obesity may be locked to an allocentric (observer view) negative memory of the body that is no longer updated by contrasting egocentric representations driven by perception. In other words, these patients may be locked to an allocentric negative representation of their body that their sensory inputs are no longer able to update even after a demanding diet and a significant weight loss.

**Objective:**

To test the brief and long-term clinical efficacy of an enhanced cognitive-behavioral therapy including a virtual reality protocol aimed at unlocking the negative memory of the body (ECT) in morbidly obese patients with binge eating disorders (BED) compared with standard cognitive behavior therapy (CBT) and an inpatient multimodal treatment (IP) on weight loss, weight loss maintenance, BED remission, and body satisfaction improvement, including psychonutritional groups, a low-calorie diet (1200 kcal/day), and physical training.

**Methods:**

90 obese (BMI>40) female patients with BED upon referral to an obesity rehabilitation center were randomly assigned to conditions (31 to ECT, 30 to CBT, and 29 to IP). Before treatment completion, 24 patients discharged themselves from hospital (4 in ECT, 10 in CBT, and 10 in IP). The remaining 66 inpatients received either 15 sessions of ECT, 15 sessions of CBT, or no additional treatment over a 5-week usual care inpatient regimen (IP). ECT and CBT treatments were administered by 3 licensed psychotherapists, and patients were blinded to conditions. At start, upon completion of the inpatient treatment, and at 1-year follow-up, patients' weight, number of binge eating episodes during the previous month, and body satisfaction were assessed by self-report questionnaires and compared across conditions. 22 patients who received all sessions did not provide follow-up data (9 in ECT, 6 in CBT, and 7 in IP).

**Results:**

Only ECT was effective at improving weight loss at 1-year follow-up. Conversely, control participants regained on average most of the weight they had lost during the inpatient program. Binge eating episodes decreased to zero during the inpatient program but were reported again in all the three groups at 1-year follow-up. However, a substantial regain was observed only in the group who received the inpatient program alone, while both ECT and CBT were successful in maintaining a low rate of monthly binge eating episodes.

**Conclusions:**

Despite study limitations, findings support the hypothesis that the integration of a VR-based treatment, aimed at both unlocking the negative memory of the body and at modifying its behavioral and emotional correlates, may improve the long-term outcome of a treatment for obese BED patients. As expected, the VR-based treatment, in comparison with the standard CBT approach, was able to better prevent weight regain but not to better manage binge eating episodes.

**Trial Registration:**

International Standard Randomized Controlled Trial Number (ISRCTN): 59019572; http://www.controlled-trials.com/ISRCTN59019572 (Archived by WebCite at http://www.webcitation.org/6GxHxAR2G)

## Introduction

Binge Eating Disorder (BED) is proposed in the *Diagnostic and Statistical Manual of Mental Disorders* (DSM IV-TR) as a new diagnostic category, requiring further research, within the spectrum of the category Eating Disorders Not Otherwise Specified (EDNOS). BED is characterized by eating a much larger amount of food than most people would consider normal in a discrete amount of time (2 hours). This is associated with a loss of control about what and how much is eaten, but without the compensatory behaviors (vomiting, use of laxatives) typical of bulimia nervosa [1]. On average, individuals suffering from BED have a high prevalence of psychiatric and medical comorbidities, as well as obesity [2]. In fact, BED may occur in up to 30% of extremely obese subjects seeking treatment at weight loss programs [3], and obesity may be observed in approximately 65-70% of people with BED [4]. Cognitive behavioral therapy (CBT) can be considered one of the better clinical approaches available to treat BED [5]. CBT has been shown to reduce binge days and episodes and to improve the psychological features of BED, such as measures of restraint, hunger, and disinhibition surrounding eating. However, CBT alone has not shown strong results in decreased weight and sustained weight reduction in the mid-term (3- and 6-month follow-ups) [2,5].

The rising prevalence of weight-related disorders and the lack of a clear solution are pushing eating disorder and obesity researchers to start collaborating across fields to address them. In particular, their effort is focused on the identification of risk factors that are shared between these weight-related disorders [6]. Apparently, unhealthful weight-control behaviors, such as fasting (going without eating for 24 hours for weight control), vomiting, or laxative abuse, are the common antecedents of both obesity and eating disorders [6-11]. For example, Neumark-Sztainer and colleagues [7] discussed the results of the Project EAT II (Eating Among Teens), a longitudinal study involving 2516 ethnically and socioeconomically diverse adolescents. They report that, 5 years later, the use of unhealthful weight-control behaviors increased the risk for binge eating with loss of control by a factor of six, for being overweight by a factor of three, and for extreme weight-control behaviors, such as the use of diet pills and self-induced vomiting by a factor between two and five. A similar result was found by Stice and colleagues [11]: in a different longitudinal study fasting was the best predictor for the future onset, 5 years later, of binge eating and bulimia nervosa. These data have an important clinical implication: the evidence that youths practicing unhealthful weight-control behaviors are at higher risk for obesity implies that prevention and treatment interventions should also focus on the causes of these behaviors. A study by Kostanski and Gullone [12] with a sample of 431 Australian pre-adolescent children (7-10 years) offers a possible interpretation: pre-adolescents as young as 7 years of age are unsatisfied with their body appearance and deliberately engage in restrictive eating behaviors. Moreover, a recent study [13] showed that in adolescents frequent self-weighing is associated with lower body satisfaction and higher rates of unhealthy and extreme weight control behaviors.

In particular, according to the allocentric lock hypothesis (ALH) [14,15], individuals with obesity and eating disorders may be locked to an allocentric (observer view) negative image schema [16] of their body that is no longer updated by contrasting egocentric representations driven by perception. In other words, these patients may be locked to a negative image of their body that perception is not able to update even after a demanding diet and a significant weight loss. In sum, they cannot win. Whatever they do to modify their real body, they will always be locked in a negative body perception that they hate. This situation usually has two effects: subjects either start more radical dietary restraint or, at the opposite end, decide to stop any form of food control and start “disinhibited” eating behaviors. The passage from a locked allocentric representation to eating or weight disorders may be explained by social influence: media and culture promote diet as the best way to improve body image satisfaction. However, the impossibility of improving body image, even after a demanding diet, locks the patient into an unsatisfying body experience.

Although the ALH is starting to be backed by research data with anorectic patients [17,18], we decided to include in a CBT approach a virtual reality (VR) protocol aimed both at unlocking the negative memory of the body [15] and at modifying its behavioral and emotional correlates within an inpatient, medically managed, intensive cognitive-behavioral BED treatment.

The purpose of this study is to evaluate the brief and long-term efficacy of the proposed approach (VR-enhanced CBT for obese inpatients with BED) in a randomized controlled trial. Outcome measures are weight, number of binge eating episodes during the previous month, and body satisfaction. We hypothesize that the VR-enhanced CBT (ECT) is more effective than standard CBT and a control condition in (1) maintaining and further improving weight loss, and (2) maintaining binge eating remission at 1-year follow-up after discharge from a multimodal medically managed inpatient program (IP). Furthermore, we hypothesize that ECT is more effective than standard CBT and a control condition in improving and maintaining body satisfaction.

The study was approved by the Ethical Committee of the Istituto Auxologico.

## Methods

### Participants and Procedures

This study was a randomized controlled trial (ISRCTN 59019572). In total, 124 consecutive patients seeking treatment at the Eating Disorder Unit of the Istituto Auxologico Italiano, Verbania, Italy, were seen for screening interviews for admission to the study. Criteria for participation included the following: (1) women aged 18-50 years, (2) who met DSM-IV-TR criteria for BED for at least 6 months prior to the beginning of the study, (3) no other concurrent severe psychiatric disturbance (psychosis, depression with suicidal risk, alcohol or drug abuse), (4) no concurrent involvement in other treatment for BED, including pharmacotherapy, (5) no concurrent medical condition not related to the disorder, and (6) written and informed consent to participate.

**Figure 1:**
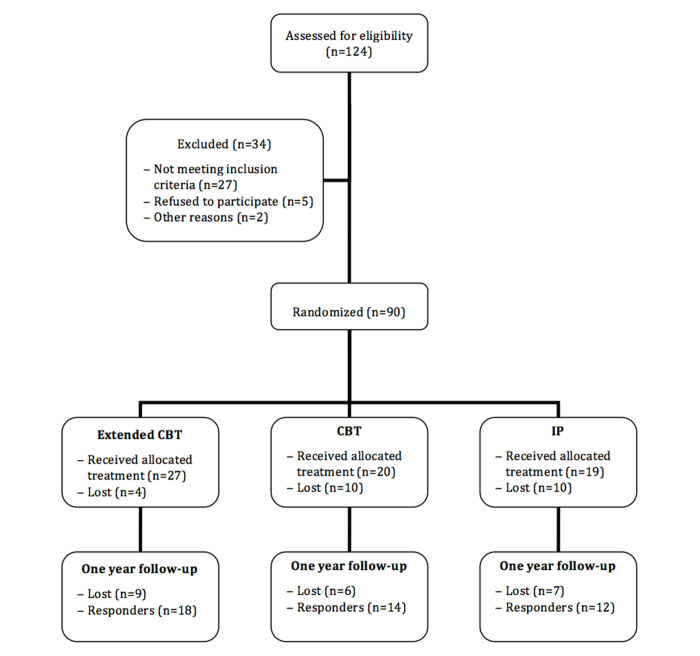
Clinical trial flowchart.

Of these, 34 either did not fulfill inclusion criteria or were excluded for other reasons (eg, time constraints). All patients meeting the inclusion criteria were then consecutively and randomly assigned to one of the three experimental conditions described below. The randomization scheme was generated by using a randomization website [19]. After allocation, 24 patients declined participation in the study (Figure 1). In total, 66 female patients (mean age 31.79±7.9 years, mean weight 106.6±17.7 kg, mean height 162±7 cm, mean BMI 40.5±5.2) entered the treatment phase (Figure 1; a detailed description of the inclusion criteria is included in the CONSORT-ehealth form available in Multimedia Appendix 1). The sample characteristics are shown in Table 1. Baseline comparisons among the three groups showed a difference only in marital status. Percentages of married patients were significantly higher in the IP group and, to a lesser extent, in the ECT group than in the CBT group.

The VR-enhanced CBT and traditional CBT (see below for treatment details) were administered by 2 licensed clinical psychologists and 1 licensed psychotherapist under the supervision of a senior licensed psychotherapist. The 3 therapists were randomized to the two treatment conditions.

**Table 1:** Baseline characteristics.

		ECT (n=27)	CBT (n=20)	IP (n=19)	*P*
Age, mean (SD), years		32.9 (8.8)	29.9 (7.95)	32.2 (6.36)	.324^a^
Weight, mean (SD), kg		103 (18.2)	106.6 (8.9)	111.6 (22.9)	.223^a^
BMI, mean (SD), kg/m^2^		39.2 (5.3)	41.1 (3.3)	41.8 (6.3)	.189^a^
**Education, n (%)**					
	University	4 (14.8)	1 (5)	2 (10.5)	.481^b^
	High school	14 (51.9)	12 (60)	14 (73.7)	
	Lower education	9 (33.3)	7 (35)	3 (15.8)	
**Marital status, n (%)**					
	Married	(44.4)	(25)	(68.4)	.026^b^

^a^Kruskall-Wallis test with Monte Carlo *P* estimation.

^b^Chi-square test with Monte Carlo *P* estimation.

#### The Integrated Multimodal Medically Managed Inpatient Program

The integrated multimodal medically managed inpatient program (IP) was the common treatment condition for all the participants. It consisted of hospital-based living for a duration of 6 weeks. Inpatients received medical, nutritional, physical, and psychological care. In particular, they maintained a low-calorie diet (tailored to patients’ needs), entered weekly nutritional groups held by dieticians, received psychological support both in individual and group settings, and undertook physical training. Participants who were allocated to this condition were considered “controls” and did not enter the following treatments.

#### Cognitive Behavior Therapy

During the inpatient program, participants allocated to this condition received 15 additional cognitive behavior therapy (CBT) sessions over 5 weeks. Therapists followed a detailed manual that outlined the contents of each session. This manual was based on the CBT approach described by Fairburn and colleagues [20,21] and by Ricca and colleagues [22]. It was developed during a year of intensive pilot work and adapted to the inpatient setting. In particular, after the first inpatient week, participants entered 5 weekly group sessions and 10 biweekly individual sessions. The first 8 individual sessions were structured according to Stage 1 of the CBT manual for binge eating. They focused on an overview of the goals of the treatment program, the use of self-monitoring records to identify high-risk situations that might trigger binge eating, support in normalizing eating patterns, and the identification of behavioral strategies for coping with high-risk situations for binge eating. The final 2 individual sessions focused on the maintenance of improvement and on relapse prevention.

The group sessions were structured according to Stage 2 of the CBT manual for binge eating. They focused on problem-solving strategies and cognitive interventions targeting concerns about body weight and shape and problematic eating. After hospital discharge, continuity of care and support through telecommunication devices (email, chat, and telephone as preferred) were offered to each patient. Contacts were not scheduled and were dependent on patients’ needs.

#### VR-Enhanced Cognitive Behavior Therapy

**Figure 2:**
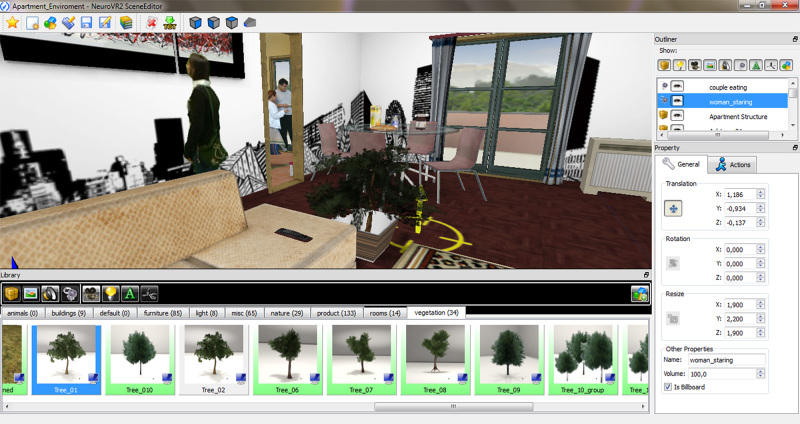
A screenshot of the NeuroVR 2 open-source software.

Like the CBT condition, participants allocated to VR-enhanced cognitive behavior therapy (ECT) received 15 additional sessions over 5 weeks. After the inpatients’ first week, participants entered 5 weekly group sessions similar to the CBT ones (focused on concerns about body weight and shape and problematic eating) and 10 biweekly VR sessions. ECT treatment was based on a detailed protocol describing the contents of each of the 15 sessions [23,24]. For the virtual reality sessions, NeuroVR open-source software was used [25-27]. NeuroVR includes 14 virtual environments used by the therapist during a 60-minute session with the patient (see Figure 2). The environments present critical situations related to the maintaining/relapse mechanisms (Home, Supermarket, Pub, Restaurant, Swimming Pool, Beach, Gymnasium) and two body image comparison areas. Through the VR experience, patients practiced both eating/emotional/relational management and general decision-making and problem-solving skills. By directly practicing these skills within the VR environment, patients were helped in developing specific strategies for avoiding and/or coping with triggering situations.

The first session was used to assess any stimuli that could elicit abnormal eating behavior. Specifically, the attention was focused on a patient’s concerns about food, eating, shape, and weight. The next 14 sessions were used to assess and modify the following.

##### Expectations and Emotions Related to Food and Weight (Functional Analysis)

The therapist helped patients to recognize why they eat and what they need to either avoid or cope with the specific emotional/behavioral triggers. This was achieved by integrating different cognitive-behavioral methods: Countering, Alternative Interpretation, Label Shifting, and Deactivating the Illness Belief.

##### Strategies Used to Cope With Difficult Interpersonal and Potential Maintenance Situations

The patient practiced old behaviors and tested new ones. This was achieved both by using the Temptation Exposure with Response Prevention [28,29] (skills training) and by working on these three empowering dimensions [30]: perceived control, perceived competence, and goal internalization (fostering the motivation*).*

##### Body Experience of the Subject

To do this, the virtual environment integrates the therapeutic methods used by Butter & Cash [31] and Wooley & Wooley [32] with the body image rescripting protocol based on ALH (see Table 2) [15,33].

In particular, we used the virtual environment in the same way as guided imagery [34] is used in the cognitive and visual/motorial approach.

After hospital discharge, continuity of care and support through telecommunication devices (email, chat, and telephone as preferred) were offered also to patients allocated to this condition. As in the previously described condition, contacts were not scheduled and were dependent on patients’ needs.

**Table 2:** The VR body image rescripting protocol (adapted from Riva, 2011).

Phase 1: Interview	During a clinical interview, the patient is asked to relive the contents of the negative body image and the situation/s in which it was created and/or reinforced (eg, “being teased by my boyfriend at home”) in as much detail as possible. The meaning of the experience for the patient was also elicited.
Phase 2: Development of the VR scene	The clinician reproduces the setting of the identified situation (eg, “the corridor of the classroom where my boyfriend teased me”) using one of the different scenes available in the free NeuroVR software.
Phase 3: Egocentric experience of the VR scene	The patient is asked to re-experience the event in VR from a first person perspective (the patient does not see his/her body in the scene) expressing and discussing his/her feelings. The patient is then asked what was needed to happen to change the feelings in a positive direction. The main cognitive techniques used in this phase, if needed, are: Countering: Once a list of distorted perceptions and cognitions is developed, the process of countering these thoughts and beliefs begins. Label Shifting: The patient first tries to identify the kinds of negative words she uses to interpret situations in her life, such as bad, terrible, obese, inferior, and hateful. The situations in which these labels are used are then listed. The patient and therapist replace each emotional label with two or more descriptive words.
Phase 4: Allocentric experience of the VR scene	The patient is asked to re-experience the event in VR from a third person perspective (the patient sees his/her body in the scene) intervening both to calm and reassure his/her virtual avatar and to counter any negative evaluation. The therapist follows the Socratic approach, for example, “What would need to happen for you to feel better? How does it look through the eyes of a third person? Is there anything you as a third person would like to do? How do the other people respond?” The main cognitive techniques used in this phase, if needed, are: Alternative Interpretation: The patient learns to stop and consider other interpretations of a situation before proceeding to the decision-making stage. Deactivating the Illness Belief: The therapist first helps the client list his/her beliefs concerning weight and eating.

### Assessment

Assessments were obtained 1 week after the start of the inpatient program, at the last week, and at 1-year follow-up (by postal mail). Height was measured with a stadiometer, and weight was assessed with the participant in lightweight clothing with shoes removed, on a balance beam scale. A single question extracted from the EDI-Symptom Checklist [35] was administered at each time-point to assess the number of binge eating episodes (with binge eating defined as the consumption of unusually large amounts of food with a subjective sense of loss of control during the last month). Data at follow-up were self-reported. The following self-report questionnaires were also administered 1 week after the start of the inpatient program, at the last week, and at 1-year follow-up (by postal mail):

- *The Italian version* [36] *of the Body Satisfaction Scale (BSS)* [37]: The scale consists of a list of 16 body parts, half involving the head (above the neck) and the other half involving the body (below the head). The subjects rate their satisfaction with each of these body parts on a 7-point scale: the higher the rating, the more dissatisfied the individual.

- *The Italian version* [38] *of the Body Image Avoidance Questionnaire (BIAQ)* [39]: The BIAQ is a 19-item self-report questionnaire on avoidance of situations that provoke concern about physical appearance, such as avoidance of tight-fitting clothes, social outings, and physical intimacy. In particular, the questionnaire measures the avoidance behaviors and grooming habits associated with negative body image.

- *Contour Drawing Rating Scale (CDRS)* [40]: This is a set of 9 male and female figures with precisely graduated increments between adjacent size. In this test, subjects rate the figures based on the following instructional protocol: (a) current size and (b) ideal size. The difference between the ratings is called the “self-ideal discrepancy score” and is considered to represent the individual’s dissatisfaction.

### Statistical Analysis

A power calculation was done to evaluate the possibility of detecting statistically significant differences both between the groups (independent measures) and within groups (repeated measures). Given the low/medium statistical power due to the relatively small number of participants, the non-normality of several distributions and the unbalanced groups, we decided to use the exact methods with Monte Carlo approximation: a series of nonparametric statistical algorithms that enable researchers to make reliable inferences when data are sparse, heavily tied or unbalanced, not normally distributed, or fail to meet any of the underlying assumptions necessary for reliable results using the standard asymptotic method [41]. The exact methods with Monte Carlo approximation used for comparisons are the Kruskal-Wallis test with post hoc analysis [42] for independent measures, the Wilcoxon rank-sum test for repeated measures, and Chi-square for categorical variables, with alpha=0.5, two-tailed. Weight data were analyzed with an intention-to-treat (ITT) analysis with nonresponders at follow-up assumed to have regained 0.3 kg per month—an assumption already used in previous studies [43,44]. Also, missing data in number of binge eating episodes during the previous month were replaced with baseline values carried forward (BCF), assuming that nonresponders had worsened. Differently, missing data at follow-up in the BSS, BIAQ, and CDRS questionnaires were not imputed because we had no assumption about the missing process. Odds ratios with confidence intervals were also calculated with respect to the percentage maintaining or improving the weight loss at follow-up for ECT and CBT treatment conditions in comparison with the inpatient program alone. Data were analyzed using SPSS 16.0.

## Results

### Inpatient Treatment Analysis

**Figure 3:**
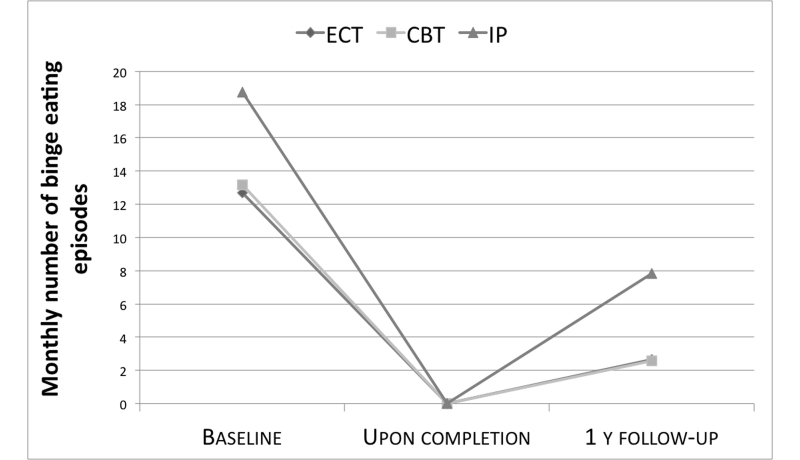
Monthly mean number of binge eating episodes at baseline, at the end of the inpatient treatment and at 1-year follow-up (dropouts at follow-up are assumed to have regained the baseline score).

Outcome data were available for all the participants at the end of treatment. Weight significantly decreased in all the three conditions (ECT: -6.17 kg, CI -7 to -5.3, *P*<.001; CBT: -7.1 kg, CI -7.9 to -6.2, *P*<.001; IP: -6.6 kg, CI -8.1 to -5.2, *P*<.001) without any significant differences between them (Table 3). This was an expected result because all the participants underwent the same 6-week medically managed inpatient treatment including a low-calorie diet and physical training (30 minutes of walking two times a week as a minimum). For the same reason, the number of binge eating episodes decreased to zero in all the three groups (Figure 3).

Body satisfaction (BSS and CDRS) significantly improved in all the groups too, with no difference across them, while body image concerns (BIAQ-Total) significantly improved only in the ECT condition. This result probably means that experiencing a medically managed inpatient treatment helped in accepting one’s own body and in decreasing dissatisfaction towards it, but only the treatment enhanced by VR exposure was effective in improving body satisfaction in relation to typical avoidance situations (Table 3).

### Follow-up Analysis

One year follow-up data were available for 66.6% (n=44) of the participants who initially entered and completed the treatment phase. Drop-out rates were similar for each group. Patients who did not respond to the follow-up call were not interviewed about their reasons for not attending the final assessments. Differences between those who did and did not respond were tested for all the variables at baseline and at the end of treatment. Statistically significant differences were found only in median numbers of baseline binge eating episodes (12 responders, 16 non-responders). With respect to the two treatment conditions, responders were only those patients who had at least one telepsychological session through telecommunication devices with the clinical psychologist or psychotherapist to whom they had been initially assigned for treatment.

ITT analysis of weight and BMI outcomes after 1-year follow-up show a different picture in comparison with the previous results (Table 3). First, statistically significant weight and BMI increases were observed in the IP group (from 105 kg to 109.3 kg; *P*<.001), while no statistically significant weight and BMI changes were found between the end of the inpatient treatment and the 1-year follow-up in CBT and ECT groups. In addition, statistically significant differences in weight and BMI median scores at follow-up were found across the three groups in favor of ECT (Table 4). In fact, only ECT was effective in further improving weight loss at 1-year follow-up.

**Table 3:** Means, standard deviations, and medians for BSS, CDRS, and BIAQ-Total at entry to the study and upon completion of the inpatient program.

		ECT (n=27^a^)			CBT^a^			IP^a^			*P* ^b^
		Mean	SD	Median	Mean	SD	Median	Mean	SD	Median	
**BSS**											
	At entry	54.85	12.8	55	60.35	8.7	62	57	12.8	57	.281
	Upon completion	45	13.9	43	52	15.5	51	47.84	13	46	.353
**CDRS**											
	At entry	1.85	0.35	1.8	2.3	1.65	1.8	1.8	0.44	1.8	.886
	Upon completion	1.58	0.36	1.5	2.02	1.69	1.6	1.6	0.35	1.5	.711
**BIAQ-Total**											
	At entry	34.4	8.5	36	33.85	5.8	33	35.53	7.16	36	.681
	Upon completion	27.2	7.23	28	31.95	6.9	32	33.1	10.26	32	.031

^a^Nonresponders are assumed to have regained 3.6 kg, 0.3 per month.

^b^Kruskall-Wallis test with Monte Carlo *P* estimation across groups at each time point.

**Table 4:** Means, standard deviations, and medians for weight and BMI at entry of the study, upon completion of the inpatient program, and at 1-year follow-up by group.

		ECT (n=27^a^)			CBT^a^			IP^a^			*P* ^b^
		Mean	SD	Median	Mean	SD	Median	Mean	SD	Median	
**Weight (kg)**											
	At entry	103	18.2	97.6	106.6	8.9	105.8	111.7	22.9	109	.223
	Upon completion	96.9	16.7	93.6	99.5	7.9	100	105	21.8	102	.251
	1-year follow-up	96	16.3	92	101	9.4	103.7	109.3	22.6	112	.032
**BMI**											
	At entry	39.2	5.3	38.1	41.1	3.3	40.8	41.8	6.3	42	.189
	Upon completion	36.9	5	36.5	38.3	3	38	39.3	5.9	40.3	.228
	1-year follow-up	36.6	5	36.2	39	3.6	39.1	40.9	6	41.5	.015

^a^Nonresponders are assumed to have regained 3.6 kg, 0.3 per month.

^b^Kruskall-Wallis test with Monte Carlo *P* estimation across groups at each time point.

**Figure 4:**
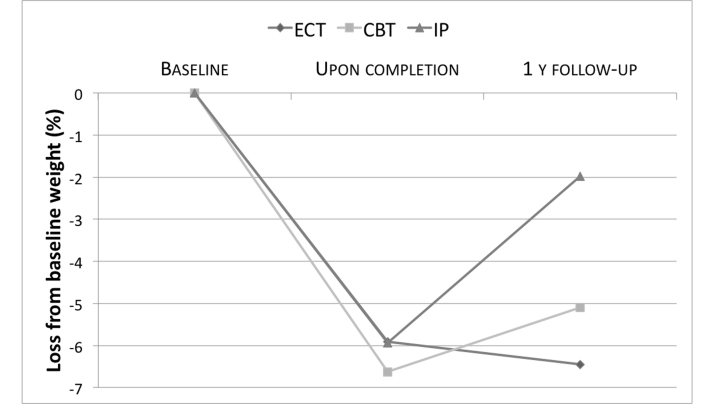
Median percent weight reduction at the end of the inpatient treatment and at 1-year follow-up (dropouts at follow-up are assumed to have regained 3.6 kg, 0.3 per month).

With respect to percentages of weight and BMI reductions at 1-year follow-up from baseline, almost significant differences emerged between the three groups (*P*=.052 for both) in favor of ECT and CBT (Figure 4). Post hoc comparisons showed a significant difference between ECT and IP (*P*=.027). Furthermore, ECT was significantly better after 1-year follow-up in improving or maintaining weight loss after treatment than IP alone. The percentages of participants who succeeded in weight maintenance or in further loss was 44.4% in ECT versus 10.5% in IP (OR 6.8, 95% CI 1.3-35.4, *P*=.014). Also CBT was significantly better after 1-year follow-up in improving or maintaining weight loss than IP alone, with 40% of participants being successful (OR 5.7, 95% CI 1.09-31.5, *P*=.035). With respect to the 5% weight loss criterion (from baseline), ITT analyses did not detect any statistically significant difference across groups, even if percentages show a trend in favor of ECT (ECT 55.6%, CBT 50%, and IP 31.6%).

Monthly number of binge eating episodes significantly increased from zero in all three groups at 1-year follow-up. ITT analysis with BCF found no statistically significant difference between the groups in follow-up median scores (Figure 3). This was largely due to the relatively great number of nonresponders we handled, assigning them the baseline scores. We decided to impute missing data in order to preserve statistical power, and we used BCF because we assumed that nonresponders had worsened.

Given that drop-out rates were not different among the three groups, we also ran a sensitivity analysis by excluding patients who did not answer the follow-up call. Results were not dissimilar and depicted the same picture we observed with imputed data.

Follow-up analyses were not performed on BSS, BIAQ, and CDRS scores because missing data were not imputed and a responders-only analysis was unfeasible due to the critical lack of information. Statistical power would have been insufficient to detect even large differences across groups and the risk of type II error would have been quite inflated.

## Discussion

We presented a randomized controlled clinical trial testing and comparing a new VR-enhanced CBT approach with standard CBT for the treatment of obese individuals with BED. Starting from the ALH [14]—suggesting that individuals with obesity may be locked to an allocentric (observer view) negative memory of the body that is no longer updated by contrasting egocentric representations driven by perception—we decided to include in the standard CBT approach to BED patients a VR-based protocol aimed at both unlocking the negative memory of the body and modifying its behavioral and emotional correlates.

We tested the efficacy of the VR-enhanced CBT on 66 female obese (BMI>40) patients with BED referred to a 6-week medically managed inpatient program (IP). Participants were randomly divided in three groups: IP (control condition), IP+CBT (15 additional sessions of group and individual CBT sessions), and IP+ECT (15 additional sessions of CBT group and VR individual sessions). We found that the inpatient program was effective in reducing weight in a sample of obese patients with BED in a relatively brief period (6 weeks) and independently from the added CBT and ECT treatments. This was a largely expected result given the highly controlled environment in which patients attended the multimodal program. The VR-enhanced CBT (ECT) delivered during the inpatient program showed to be effective in the long term. In fact, ECT proved superior to the inpatient program alone in preventing weight regain and in further improving the weight loss after 1-year follow-up. Also, standard CBT was more effective than the inpatient program alone in preventing weight regain. However, follow-up scores with nonresponders assumed to have regained 3.6 kg (0.3 per month) show a significant difference in favor of ECT. Indeed, only ECT was effective in further improving weight loss at 1-year follow-up. On the contrary, control participants regained on average most of the weight they had lost during the inpatient program.

Binge eating episodes decreased to zero during the inpatient program (because of the same reasons explained for weight reduction) but were reported again in all three groups at 1-year follow-up. However, in the ITT analysis with BCF, a substantial regain was observed only in the group who received the inpatient program alone, while both ECT and CBT were successful in maintaining a low rate of monthly binge eating episodes (Figure 3).

### Limitations

Some study limitations deserve attention. The first and most important one is the medium-high rate of nonresponders (33.4%); thus, our follow-up results have to be considered with caution. In order to preserve the ITT principle and statistical power, we handled missing data by imputation, assuming that patients who did not provide data at 1-year follow-up had worsened. With respect to weight, we imputed data according to a plausible empirical rule already used in previous studies [43,44], while missing data in a number of binge eating episodes were handled by an imputation method (BCF) that deserves more critical consideration [45]. Second, the CBT treatment used in this study is a reduced (6-week) version of the Oxford outpatient protocol [20,21]. Obviously, major changes have been made to the original 44-week format, so we cannot compare our results with ones obtained in studies that implemented the original protocol. Third, no other psychosocial variable was measured beyond body image satisfaction. As a reviewer suggested, measures of emotional and social functioning would have added further information on the efficacy of the ECT treatment. We agree and hope that future studies on this novel VR-enhanced treatment will include further measures of psychological, emotional, and social variables that can act as outcomes as well as mediators or moderators of treatment efficacy.

### Conclusion

The rising prevalence of weight-related disorders and the lack of a clear solution are pushing eating disorder and obesity researchers to collaborate. In particular, their efforts have identified a critical risk factor: unhealthful weight-control behaviors, such as fasting, vomiting, or laxative abuse, are the common antecedents of both obesity and binge eating. But why do these subjects decide to start such radical weight-control behaviors? The answer underlined both by clinical practice and by research studies is simple: because subjects do not like their bodies.

Given the importance of body image satisfaction for the quality of life of these patients, these findings argue for the potential benefits of treatment strategies for improving body satisfaction. Unfortunately, this vision is not shared by the existing treatment protocols. As noted by Rosen 15 years ago, only a third of cognitive-behavioral treatments assessed in his review addressed body image specifically [46]. The situation still has not changed very much. And when it happens, the focus is more on shape concerns and over-evaluation than on the experience and perception of a fat body [47].

Recently the ALH [14,15] offered a clear link between a negative body image and the etiology of obesity and eating disorders, ie, patients may be locked to an allocentric (observer view) negative image schema of their body that is not updated by contrasting egocentric representations driven by perception. In other words, they cannot win: whatever they do to modify their real body, they will always be present in a virtual body that they hate. This situation has usually two effects: subjects either start more radical dietary restraint or, the opposite, they decide to stop any form of food control and start “disinhibited” eating behaviors.

In this study, we tested a modified CBT approach that included a VR-based treatment to target this issue. VR can be considered an “embodied technology” for its effects on body perceptions [48]. On one side, different authors showed that is possible to use VR both to induce illusory perceptions, eg, a fake limb [49], by altering the normal association between touch and its visual correlate. It is even possible to generate a body transfer illusion [50]: Slater and colleagues substituted the experience of male subjects’ own bodies with a life-sized virtual human female body. On the other side, it is also possible to use VR to improve body image [51,52], even in patients with eating disorders [23,53,54] or obesity [55,56]

Despite the study limitations discussed above, findings support the hypothesis that the integration of a VR-based treatment, aimed at both unlocking the negative memory of the body and at modifying its behavioral and emotional correlates, may improve the long-term outcome of a treatment for obese BED patients. This result is in line with the recent review [57] about the use of VR for the treatment of body image in eating disorders. The authors concluded their paper with the following words: “VR-based therapies seem to be especially suitable for improving body image both in ED patients and in subclinical samples…All these studies showed significantly greater improvement in measures related with body image when the VR component was added.” (p. 9). The ALH discussed in this paper may be a possible explanation for this result. However, due to the integrated structure of the VR intervention and to the assessment tools used in the trial, we are not able to verify a possible direct effect of the proposed treatment on the allocentric lock (only indirect through a better body satisfaction). So, future studies are needed to further explore and verify this specific hypothesis.

As expected, the VR-based treatment, in comparison with the standard CBT approach, was able to better prevent weight regain but not to better manage binge eating episodes. This is because both CBT and ECT shared the same protocol on problem-solving strategies and cognitive interventions targeting problematic eating.

Future research should focus on using meta-analysis techniques in order to test the real strength of effects found in the clinical studies, and on conducting more controlled studies comparing VR-based treatments with traditional ones. To reach this goal, the “Laboratorio de Enseñanza Virtual y Ciberpsicología” at the School of Psychology of the Universidad Nacional Autonoma de Mexico, in cooperation with the Obesity Unit of the Medica Sur Hospital in Mexico City, have recently started a controlled clinical trial [58,59].

## Multimedia Appendix 1

CONSORT-EHEALTH checklist V1.6.2 [59].

## Abbreviations

ALH: allocentric lock hypothesis
BCF: baseline values carried forward
BED: binge eating disorders
BIAQ: Body Image Avoidance Questionnaire
BSS: Body Satisfaction Scale
CBT: cognitive behavioral therapy
CDRS: Contour Drawing Rating Scale
ECT: VR-enhanced cognitive behavior therapy
IP: integrated multimodal medically managed inpatient program
ITT: intention-to-treat
VR: virtual reality
