# Supplementary material for: Virtual Reality for Enhancing the Cognitive Behavioral Treatment of Obesity With Binge Eating Disorder: Randomized Controlled Study With One-Year Follow-up
Source: J Med Internet Res. 2013 Jun 12;15(6):e113. doi: 10.2196/jmir.2441 (PMC3713949; doi:10.2196/jmir.2441)
Supplement: Supplementary file 1 [file jmir_v15i6e113_app1.pdf]

CONSORT-EHEALTH Checklist V1.6.2 Report

(based on CONSORT-EHEALTH V1.6), available at [http://tinyurl.com/consort-ehealth-v1-6].

2441

Date completed

3/12/2013 13:33:26

by

Gian Mauro Manzoni

Virtual Reality for Enhancing the Cognitive Behavioral Treatment of Obesity with Binge Eating Disorder: A Randomized Controlled Trial with One-Year Follow-up

TITLE

1a-i) Identify the mode of delivery in the title

"Virtual Reality for Enhancing the Cognitive Behavioral Treatment of Obesity with Binge Eating Disorder"

1a-ii) Non-web-based components or important co-interventions in title

Not applicable to our trial

1a-iii) Primary condition or target group in the title

"Virtual Reality for Enhancing the Cognitive Behavioral Treatment of Obesity with Binge Eating Disorder"

ABSTRACT

1b-i) Key features/functionalities/components of the intervention and comparator in the METHODS section of the ABSTRACT

"To test the brief and long-term clinical efficacy of an enhanced cognitive behavioural therapy including a virtual reality ... - compared with standard cognitive behavior therapy (CBT) and an integrated inpatient multi-modal treatment (IPT)"

1b-ii) Level of human involvement in the METHODS section of the ABSTRACT

"ECT and CBT treatments were administered by three licensed psychotherapists".

1b-iii) Open vs. closed, web-based (self-assessment) vs. face-to-face assessments in the METHODS section of the ABSTRACT

"Sixty six female obese (BMI>40) inpatients with BED were recruited consecutively upon referral to an obesity rehabilitation center".
"weight, number of binge eating episodes during the previous month and body satisfaction were assessed by self-report questionnaires".
"patients were blinded to conditions"

1b-iv) RESULTS section in abstract must contain use data

"Ninety female patients were randomly assigned to conditions (31 to ECT, 30 to CBT and 29 to IP). Before treatment completion, 24 patients discharged themselves from hospital (4 in ECT, 10 in CBT and 10 in IP) and 22 patients who received all sessions did not provide follow-up data (9 in ECT, 6 in CBT and 7 in IP)".

1b-v) CONCLUSIONS/DISCUSSION in abstract for negative trials

"findings support the hypothesis that the integration of a VR-based treatment, aimed at unlocking the negative memory of the body, may improve the long-term outcome of an obesity treatment"

INTRODUCTION

2a-i) Problem and the type of system/solution

"Cognitive behavior therapy can be considered one of the better available clinical approach for BED [5]. ... . However, CBT alone has not strongly resulted in decreased weight and sustained weight reduction in the mid-term".
"In our opinion, a new promising cognitive-behavioral approach targeting BED and its frequently associated obesity may come from a current scientific interest in linking the etiology of obesity to unhealthful weight-control behaviors (fasting, vomiting or laxative abuse) induced by a negative experience of the body".
"we decided to include in a CBT approach a Virtual Reality (VR) protocol aimed at unlocking the negative memory of the body within an inpatient, medically-managed intensive cognitive-behavioral obesity treatment".

2a-ii) Scientific background, rationale: What is known about the (type of) system

"according to the Allocentric Lock Hypothesis, individuals with obesity and eating disorders may be locked to an allocentric negative memory of the body that is no longer updated by contrasting egocentric representations driven by perception. .... we decided to test the hypothesis with obese BED. In particular, we decided to include in a CBT approach a Virtual Reality (VR) protocol aimed at unlocking the negative memory of the body..".

METHODS

3a) CONSORT: Description of trial design (such as parallel, factorial) including allocation ratio

"The purpose of this study is to evaluate the brief and long-term efficacy of the proposed approach (VR-enhanced CBT for obese inpatients with BED) in a randomized controlled trial. ... . We hypothesize that the VR-enhanced CBT (ECT) is more effective than standard CBT and a control condition in: 1- maintaining and further improving weight loss; 2- maintaining binge eating remission at 1-year follow-up after discharge from a multimodal medically-managed inpatient program. Furthermore, we hypothesize that ECT is more effective than standard CBT and a control condition in improving and maintaining body satisfaction."

3b) CONSORT: Important changes to methods after trial commencement (such as eligibility criteria), with reasons

Not applicable to our trial because no change was made to methods after trial commencement.

3b-i) Bug fixes, Downtimes, Content Changes

No change was made to the VR systems or contents due to failures, downtimes or staff changes.

4a) CONSORT: Eligibility criteria for participants

"Criteria for participation included the following: (1) women aged 18–50 years; (2) who met DSM-IV-TR criteria for BED for at least 6 months prior to the beginning of the study; (3) no other concurrent severe psychiatric disturbance (psychosis, depression with suicidal risk, alcohol or drug abuse); (4) no concurrent involvement in other treatment for BED, including pharmacotherapy; (5) no concurrent medical condition not related to the disorder; (6) written and informed consent to participate."

4a-i) Computer / Internet literacy
In our trial, no computer or other ICT literacy was needed by patients in order to have the VR treatment.
4a-ii) Open vs. closed, web-based vs. face-to-face assessments:
"Sixty six female obese (BMI>40) inpatients with BED were recruited consecutively upon referral to an obesity rehabilitation center".
"self-report questionnaires were administered face-to-face in the clinical center one week after the start of the inpatient program and at the last week. Data at 1-year follow-up were collected by sending self-report questionnaires to patients' home by postal mail."
4a-iii) Information giving during recruitment
Patients were briefed by one of the psychotherapists and read the informed consent form before signing and accepting to participate.
4b) CONSORT: Settings and locations where the data were collected
"One hundred and twenty four consecutive patients seeking treatment at the Eating Disorder Unit of the Istituto Auxologico Italiano, Verbania, Italy, were seen for screening interviews for admission to the study."
4b-i) Report if outcomes were (self-)assessed through online questionnaires
"self-report questionnaires were administered face-to-face in the clinical center one week after the start of the inpatient program and at the last week. Data at 1-year follow-up were collected by sending self-report questionnaires to patients' home by postal mail."
4b-ii) Report how institutional affiliations are displayed
Not applicable to our trial because no e-health media was used.
5) CONSORT: Describe the interventions for each group with sufficient details to allow replication, including how and when they were actually administered
5-i) Mention names, credential, affiliations of the developers, sponsors, and owners
The software NeuroVR was developed by Virtual Reality & Multimedia Park, Turin, Italy, under the clinical supervision of Istituto Auxologico Italiano. We added this statement in the Conflict of Interest.
5-ii) Describe the history/development process
The software underwent different phases of development and testing that are detailed in the following papers:

Riva, G., Gaggioli, A., Villani, D., Preziosa, A., Morganti, F., Corsi, R., et al. (2007). NeuroVR: an open source virtual reality platform for clinical psychology and behavioral neurosciences. Studies in Health Technology and Informatics, 125, 394-399.
Riva, G., Carelli, L., Gaggioli, A., Gorini, A., Vigna, C., Corsi, R., et al. (2009). NeuroVR 1.5 - a free virtual reality platform for the assessment and treatment in clinical psychology and neuroscience. Stud Health Technol Inform, 142, 268-270.
Riva, G., Carelli, L., Gaggioli, A., Gorini, A., Vigna, C., Algeri, D., et al. (2009). NeuroVR 1.5 in Practice: Actual Clinical Applications of the Open Source VR System. Stud Health Technol Inform, 144, 57-60.
Riva, G., Gaggioli, A., Grassi, A., Raspelli, S., Cipresso, P., Pallavicini, F., et al. (2011). NeuroVR 2 - A Free Virtual Reality Platform for the Assessment and Treatment in Behavioral Health Care. Stud Health Technol Inform, 163, 493-495.
5-iii) Revisions and updating
No significant revision or updating was made during the trial
5-iv) Quality assurance methods
Not applicable to this trial.
5-v) Ensure replicability by publishing the source code, and/or providing screenshots/screen-capture video, and/or providing flowcharts of the algorithms used
"For the virtual reality sessions, the NeuroVR open source software (http://www.neurovr.org) was used."
5-vi) Digital preservation
"For the virtual reality sessions, the NeuroVR open source software (http://www.neurovr.org) was used."
5-vii) Access
Participants received treatments during inpatient hospitalization. They had not to pay and they had not to be a member of specific group.
"For the virtual reality sessions, the NeuroVR open source software (http://www.neurovr.org) was used."
5-viii) Mode of delivery, features/functionalities/components of the intervention and comparator, and the theoretical framework
"During the inpatient program, participants allocated to CBT condition received 15 additional CBT sessions over 5 weeks. Therapists followed a detailed manual that outlined the contents of each session. This manual was based on the CBT approach described by Fairburn and colleagues and by Ricca and colleagues. It was developed during a year of intensive pilot work and adapted to the inpatient setting. In particular, after the first inpatient week, participants entered five weekly group sessions and 10 biweekly individual sessions.
After hospital discharge, continuity of care and support through telecommunication devices (e-mail, chat and telephone as preferred) were offered to each patients. Contacts were not scheduled and were dependent only of patient’s needs."
"Like the CBT condition, participants allocated to ECT treatment received 15 additional sessions over 5 weeks. After the first inpatients week, participants entered 5 weekly group sessions similar to the CBT ones and 10 biweekly virtual reality sessions. ECT treatment was based on a detailed protocol describing the contents of each of the 15 sessions. For the virtual reality sessions, the NeuroVR open source software (http://www.neurovr.org) was used. NeuroVR includes 14 virtual environments used by the therapist during a 60-min session with the patient. The environments present critical situations related to the maintaining/relapse mechanisms (Home, Supermarket, Pub, Restaurant, Swimming Pool, Beach, Gymnasium) and two body image comparison areas. Through the VR experience, patients practice both eating/emotional/relational management and general decision-making and problem-solving skills. By directly practicing these skills within the VR environment, patients are helped in developing specific strategies for avoiding and/or coping with triggering situations."
5-ix) Describe use parameters
Not applicable to our trial because treatments were administered by psychotherapists according to a fixed schedule.
5-x) Clarify the level of human involvement
"The VR-enhanced CBT and traditional CBT were administered by two licensed clinical psychologists and one licensed psychotherapist under the supervision of a senior licensed psychotherapist. The three therapists were randomized to the two treatment conditions."
5-xi) Report any prompts/reminders used
Not applicable to our trial because no prompt/reminder was necessary.
5-xii) Describe any co-interventions (incl. training/support)
"The integrated multimodal medically-managed inpatient program (IP).
This was the common treatment condition for all the participants. It consists in a hospital-based living that lasts for 6 weeks. Inpatients receive medical, nutritional, physical and psychological care. In particular, they maintain a low-calorie diet (tailored on patient’s need), enter weekly nutritional groups held by dieticians, receive psychological support both in individual and group setting, and undertake physical training. Participants who were allocated to this condition were considered “controls” and did not enter the following treatments."
6a) CONSORT: Completely defined pre-specified primary and secondary outcome measures, including how and when they were assessed

"We hypothesize that the VR-enhanced CBT (ECT) is more effective than standard CBT and a control condition in: 1- maintaining and further improving weight loss; 2- maintaining binge eating remission at 1-year follow-up after discharge from a multimodal medically-managed inpatient program. Furthermore, we hypothesize that ECT is more effective than standard CBT and a control condition in improving and maintaining body satisfaction."
"Assessments were obtained one week after the start of the inpatient program, at the last week and at 1-year follow-up (by postal mail). Height was measured with a stadiometer and weight was assessed with the participant in lightweight clothing with shoes removed, on a balance beam scale. A single question extracted from the EDI-Symptom Checklist was administered at each time-points to assess the number of binge eating episodes (binge eating defined as the consumption of unusually large amounts of food with a subjective sense of loss of control during the last month). Data at follow-up were self-reported. The following self-report questionnaires were also administered one week after the start of the inpatient program, at the last week and at 1-year follow-up (by postal mail)"

6a-i) Online questionnaires: describe if they were validated for online use and apply CHERRIES items to describe how the questionnaires were designed/deployed

Not applicable to our trial because no online questionnaire was used.

6a-ii) Describe whether and how "use" (including intensity of use/dosage) was defined/measured/monitored

Not applicable to our study because no use/adoption metric was necessary.

6a-iii) Describe whether, how, and when qualitative feedback from participants was obtained

No qualitative feedback from participants was obtained.

6b) CONSORT: Any changes to trial outcomes after the trial commenced, with reasons

No change was made to trial outcomes after the trial commenced

7a) CONSORT: How sample size was determined

7a-i) Describe whether and how expected attrition was taken into account when calculating the sample size

Possible attrition was not taken into account when calculating the sample size

7b) CONSORT: When applicable, explanation of any interim analyses and stopping guidelines

Not applicable to our trial because no interim analysis was performed and no stopping rule was defined.

8a) CONSORT: Method used to generate the random allocation sequence

The randomization scheme was generated by using the Web site Randomization.com (www. randomization.com).

8b) CONSORT: Type of randomisation; details of any restriction (such as blocking and block size)

Simple randomisation was used.

9) CONSORT: Mechanism used to implement the random allocation sequence (such as sequentially numbered containers), describing any steps taken to conceal the sequence until interventions were assigned

"Included patients were randomized to the three conditions soon after baseline assessment using pre-prepared closed envelopes containing the allocation notes. The simple randomization sequence was generated by an independent research assistant who prepared also the envelopes containing the allocation notes."

10) CONSORT: Who generated the random allocation sequence, who enrolled participants, and who assigned participants to interventions

"The simple randomization sequence was generated by an independent research assistant".
"Patients were enrolled by psychotherapists and assigned to conditions by the same independent research assistant who prepared the envelopes"

11a) CONSORT: Blinding - If done, who was blinded after assignment to interventions (for example, participants, care providers, those assessing outcomes) and how

11a-i) Specify who was blinded, and who wasn't

Patients were blinded to conditions, while psychotherapists weren't.

11a-ii) Discuss e.g., whether participants knew which intervention was the "intervention of interest" and which one was the "comparator"

"Participants did not know which condition was the treatment of interest and which one was the comparator".

11b) CONSORT: If relevant, description of the similarity of interventions

"Like the CBT condition, participants allocated to VR-enhanced treatment received 15 additional sessions over 5 weeks. After the first inpatients week, participants entered 5 weekly group sessions similar to the CBT ones and 10 biweekly virtual reality sessions. ECT treatment was based on a detailed protocol describing the contents of each of the 15 sessions."

12a) CONSORT: Statistical methods used to compare groups for primary and secondary outcomes

"The exact methods with Monte Carlo approximation used for comparisons are the Kruskal-Wallis test with post hoc analysis for independent measures, the Wilcoxon rank-sum test for repeated measures and Chi-square for categorical variables."

12a-i) Imputation techniques to deal with attrition / missing values

"Weight data were analyzed with an intention-to-treat (ITT) analysis with non-responders at follow-up assumed to have regained 0,3 kg per month, an assumption already used in previous studies. Also missing data in number of binge eating episodes during the previous month were replaced with baseline values carried forward (BCF), assuming that non-responders had worsened. Differently, missing data at follow-up in the BSS, BIAQ and CDRS questionnaires were not imputed because we had no assumption about the missing process."

12b) CONSORT: Methods for additional analyses, such as subgroup analyses and adjusted analyses

No additional analysis was performed.

RESULTS

13a) CONSORT: For each group, the numbers of participants who were randomly assigned, received intended treatment, and were analysed for the primary outcome

"Ninety female patients were randomly assigned to conditions (31 to ECT, 30 to CBT and 29 to IP). Before treatment completion, 24 patients discharged themselves from hospital (4 in ECT, 10 in CBT and 10 in IP) and 22 patients who received all sessions did not provide follow-up data (9 in ECT, 6 in CBT and 7 in IP)."
"ECT and CBT treatments were administered by three licensed psychotherapists who treated 22 patients each".
"Outcome data were available for 66 patients at the end of treatment."
"One year follow-up data were available for 66,6% (n=44) of the participants who completed the treatment phase. Attrition rates were similar for each group".

13b) CONSORT: For each group, losses and exclusions after randomisation, together with reasons

"After allocation to treatment conditions, 24 patients interrupted participation and provided no reason for their decision"
"Patients who did not respond to the follow-up call were not interviewed about their reasons for not attending the final assessments."

13b-i) Attrition diagram

An attrition diagram was included in the text.

14a) CONSORT: Dates defining the periods of recruitment and follow-up

The trial lasted 18 months, plus a 12-month follow-up. The recruitment phase ended at month 16th.

14a-i) Indicate if critical "secular events" fell into the study period

No "secular events" fell into the study perdiod.

14b) CONSORT: Why the trial ended or was stopped (early)
The trial did not end or was stopped early.
15) CONSORT: A table showing baseline demographic and clinical characteristics for each group
A table showing baseline demographic and clinical characteristics for each group was included.
15-i) Report demographics associated with digital divide issues
Not applicable to our trial because digital divide was not an issue.
16a) CONSORT: For each group, number of participants (denominator) included in each analysis and whether the analysis was by original assigned groups
16-i) Report multiple “denominators” and provide definitions
"One year follow-up data were available for 66,6% (n=44) of the participants who initially entered and completed the treatment phase. Attrition rates were similar for each group".
An intention-to-treat (ITT) analysis approach was used.
16-ii) Primary analysis should be intent-to-treat
An intention-to-treat (ITT) analysis approach was used.
17a) CONSORT: For each primary and secondary outcome, results for each group, and the estimated effect size and its precision (such as 95% confidence interval)
Non-parametric analyses were used and no parametric effect size was calculated.
17a-i) Presentation of process outcomes such as metrics of use and intensity of use
Not applicable to our trial because no dose/exposure metric was used.
17b) CONSORT: For binary outcomes, presentation of both absolute and relative effect sizes is recommended
"The percentages of participants who succeeded in weight maintenance or in further loss was 44.4% in ECT versus 10.5% in IP (Odds ratio 6.8, 95% CI 1.3 to 35.4, p=0.014). Also CBT was significantly better after 1 year follow-up in improving or maintaining weight loss than IP alone, with 40% of participants being successful (Odds ratio 5.7, 95% CI 1.09 to 31.5, p=0.035). With respect to the 5% weight loss criterion (from baseline), intention-to-treat analyses did not detect any statistically significant difference across groups, even if percentages show a trend in favour of ECT (ECT 55.6%, CBT 50% and IP 31.6%).
18) CONSORT: Results of any other analyses performed, including subgroup analyses and adjusted analyses, distinguishing pre-specified from exploratory
According to one of referees' suggestions, a sub-group analysis comparing only responders to follow-up was performed.
18-i) Subgroup analysis of comparing only users
"Given that drop-out rates were not different among the three groups, we ran also a sensitivity analysis by excluding patients who did not answer the follow-up call. Results were not dissimilar and depicted the same picture we observed with imputed data."
19) CONSORT: All important harms or unintended effects in each group
No harm or unintended effect was expected and observed.
19-i) Include privacy breaches, technical problems
Not applicable to our trial because no privacy breaches or technical problems were expected.
19-ii) Include qualitative feedback from participants or observations from staff/researchers
No qualitative feedback from participants or observations from staff/researchers was collected.

DISCUSSION

20) CONSORT: Trial limitations, addressing sources of potential bias, imprecision, multiplicity of analyses
20-i) Typical limitations in ehealth trials
Study limitations were fully addressed.
21) CONSORT: Generalisability (external validity, applicability) of the trial findings
21-i) Generalizability to other populations
The interventions are fully applicable in any other clinical unit for the treatment of obese people with BED.
Trial findings can be generalized only to inpatient clinical unit.
21-ii) Discuss if there were elements in the RCT that would be different in a routine application setting
Not fully applicable to this trial. In both experimental and comparison groups the therapists use the same clinical approach.
22) CONSORT: Interpretation consistent with results, balancing benefits and harms, and considering other relevant evidence
22-i) Restate study questions and summarize the answers suggested by the data, starting with primary outcomes and process outcomes (use)
Study questions were restated and answers were summarized.
22-ii) Highlight unanswered new questions, suggest future research
"no other psychosocial variable was measured beyond body image satisfaction. As a reviewer suggested, measures of emotional and social functioning would have added further information on the efficacy of the ECT treatment. We agree with him and hope that future studies on this novel VR-enhanced treatment will include further measures of psychological, emotional and social variables that can act as outcomes as well as mediators or moderators of treatment efficacy."

Other information

23) CONSORT: Registration number and name of trial registry
"Trial Registration: controlled-trials.com ISRCTN59019572 "
24) CONSORT: Where the full trial protocol can be accessed, if available
www.controlled-trials.com
ISRCTN59019572
25) CONSORT: Sources of funding and other support (such as supply of drugs), role of funders
The present work was supported by the Commission of the European Communities (CEC), through its IST programme (Project “VEPSY Updated” and Project “INTREPID”) and by the Italian MIUR FIRB programme (Project “IVT2010”).
X26-i) Comment on ethics committee approval
"The study was approved by the Ethical Committee of the Istituto Auxologico Italiano."
x26-ii) Outline informed consent procedures
The informed consent form was signed directly by patients, face-to-face with the psychotherapists.
A copy of the informed consent form is in the appendix.
X26-iii) Safety and security procedures
Not applicable to our trial because no e-media involving personal data was used.
X27-i) State the relation of the study team towards the system being evaluated

The technical developers and the sponsor of the virtual reality tools were not involved in the clinical trials. However, the clinical teams involved in the trial helped to define the contents of the virtual experience and defined the clinical protocol.
